# Supplementary material for: Under-Five Mortality and Associated Factors: Evidence from the Nepal Demographic and Health Survey (2001–2016)
Source: Int J Environ Res Public Health. 2019 Apr 8;16(7):1241. doi: 10.3390/ijerph16071241 (PMC6479835; doi:10.3390/ijerph16071241)
Supplement: Supplementary file 1 [file ijerph-16-01241-s001.zip › supplementary table 1.docx]

Supplementary table 1: aHR and 95% Confidence Interval (CI) for factors associated with neonatal, post-neonatal, and infant mortality in Nepal, 2001-2016 (n=15,750)

|  | Neonatal mortality | | | Post-neonatal mortality | | | Infant mortality | | |
| --- | --- | --- | --- | --- | --- | --- | --- | --- | --- |
| Study Variable | Model 1^a^ | Model 2^b^ | Model 3^c^ | Model 1^a^ | Model 2^b^ | Model 3^c^ | Model 1^a^ | Model 2^b^ | Model 3^c^ |
| Year of survey |  |  |  |  |  |  |  |  |  |
| 2001 | 1.00 | 1.00 | 1.00 | 1.00 |  |  | 1.00 |  |  |
| 2006 | 0.77(0.53, 1.13) | 0.85(0.60, 1.21) | 0.85(0.60, 1.21) | 0.87(0.53, 1.41) |  |  | 0.80(0.60, 1.08) |  |  |
| 2011 | 0.82(0.56, 1.19) | 1.01(0.71, 1.45) | 1.01(0.71, 1.45) | 0.55(0.33, 0.91)* |  |  | 0.72(0.53, 0.98)* |  |  |
| 2016 | 0.44(0.28, 0.67)** | 0.60(0.40, 0.92)* | 0.60(0.40, 0.92)* | 0.65(0.38, 1.13) |  |  | 0.52(0.37, 0.73)** |  |  |
| Ecological zone |  |  |  |  |  |  |  |  |  |
| Terai |  |  |  | 1.00 | 1.00 | 1.00 | 1.00 | 1.00 | 1.00 |
| Hill |  |  |  | 0.90(0.59, 1.35) | 0.89(0.60, 1.33) | 0.89(0.60, 1.33) | 0.97(0.76, 1.23) | 1.18(0.89, 1.55) | 1.18(0.89, 1.55) |
| Mountain |  |  |  | 2.00(1.26, 3.18)* | 1.70(1.07, 2.68)* | 1.70(1.07, 2.68)* | 1.54(1.15, 2.06)* | 1.47(1.08, 2.01)* | 1.47(1.08, 2.01)* |
| Religion |  |  |  |  |  |  |  |  |  |
| Buddhist |  |  |  |  | 1.00 | 1.00 |  |  |  |
| Hindu |  |  |  |  | 2.43(0.99, 5.93) | 2.43(0.99, 5.93) |  |  |  |
| Others |  |  |  |  | 3.77(1.30, 10.92)* | 3.77(1.30, 10.92)* |  |  |  |
| Ethnicity |  |  |  |  |  |  |  |  |  |
| Brahmin/chettri |  |  |  |  |  |  |  | 1.00 | 1.00 |
| Dalit |  |  |  |  |  |  |  | 1.16(0.83, 1.62) | 1.16(0.83, 1.62) |
| Janajati |  |  |  |  |  |  |  | 0.86(0.64, 1.15) | 0.86(0.64, 1.15) |
| Madhesi |  |  |  |  |  |  |  | 1.76(1.21, 2.55)* | 1.76(1.21, 2.55)* |
| Mother education |  |  |  |  |  |  |  |  |  |
| Secondary or higher |  |  |  |  |  |  |  | 1.00 | 1.00 |
| Primary |  |  |  |  |  |  |  | 1.52(1.01, 2.30)* | 1.52(1.01, 2.30)* |
| No education |  |  |  |  |  |  |  | 1.87(1.31, 2.68)* | 1.87(1.31, 2.68)* |
| Mother's literacy level |  |  |  |  |  |  |  |  |  |
| Can read |  | 1.00 | 1.00 |  |  |  |  |  |  |
| Cannot read |  | 1.80(1.31, 2.47)** | 1.80(1.31, 2.47)** |  |  |  |  |  |  |
| Mother occupation |  |  |  |  |  |  |  |  |  |
| Not working |  |  |  |  | 1.00 | 1.00 |  | 1.00 | 1.00 |
| Agriculture |  |  |  |  | 2.05(1.18, 3.57)* | 2.05(1.18, 3.57)* |  | 1.50(1.10, 2.05)* | 1.50(1.10, 2.05)* |
| Skilled/professional |  |  |  |  | 1.95(0.85, 4.43) | 1.95(0.85, 4.43) |  | 2.00(1.29, 3.10)* | 2.00(1.29, 3.10)* |
| Mother's age |  |  |  |  |  |  |  |  |  |
| 40-49 |  | 1.00 | 1.00 |  | 1.00 | 1.00 |  | 1.00 | 1.00 |
| 30-39 |  | 1.35(0.76, 2.38) | 1.35(0.76, 2.38) |  | 2.02(0.99, 4.14) | 2.02(0.99, 4.14) |  | 1.54(0.98, 2.42) | 1.54(0.98, 2.42) |
| 20-29 |  | 1.57(0.90, 2.74) | 1.57(0.90, 2.74) |  | 2.94(1.25, 6.96)* | 2.94(1.25, 6.96)* |  | 1.95(1.22, 3.12)* | 1.95(1.22, 3.12)* |
| <20 |  | 2.17(1.04(4.53)* | 2.17(1.04(4.53)* |  | 4.47(1.54, 12.97)* | 4.47(1.54, 12.97)* |  | 2.75(1.49, 5.10)* | 2.75(1.49, 5.10)* |
| Birth rank and birth interval |  |  |  |  |  |  |  |  |  |
| 2nd/3rd birth rank, >2 years |  | 1.00 | 1.00 |  | 1.00 | 1.00 |  | 1.00 | 1.00 |
| 1st child |  | 3.07(1.86, 5.04)** | 3.07(1.86, 5.04)** |  | 2.30(1.15, 4.62)* | 2.30(1.15, 4.62)* |  | 2.97(1.97, 4.47)** | 2.97(1.97, 4.47)** |
| 2nd/3rd child, interval ≤2 years |  | 1.26(0.76, 2.07) | 1.26(0.76, 2.07) |  | 1.00(0.52, 1.91) | 1.00(0.52, 1.91) |  | 1.19(0.79, 1.80) | 1.19(0.79, 1.80) |
| 4th/higher child, interval >2 years |  | 0.30(0.18, 0.52)** | 0.30(0.18, 0.52)** |  | 0.55(0.27, 1.10) | 0.55(0.27, 1.10) |  | 0.37(0.24, 0.56)** | 0.37(0.24, 0.56)** |
| 4th/higher child, interval ≤ 2 years |  | 0.64(0.36, 1.15) | 0.64(0.36, 1.15) |  | 0.51(0.25, 1.03) | 0.51(0.25, 1.03) |  | 0.59(0.37, 0.93)* | 0.59(0.37, 0.93)* |
| Previous Death of a child |  |  |  |  |  |  |  |  |  |
| No |  | 1.00 | 1.00 |  | 1.00 | 1.00 |  | 1.00 | 1.00 |
| Yes |  | 18.75(12.28, 28.63)** | 18.75(12.28, 28.63)** |  | 14.27(7.81, 26.07)** | 14.27(7.81, 26.07)** |  | 17.05(12.24, 23.74)** | 17.05(12.24, 23.74)** |

aHR: adjusted Hazard Ratio; **: p<0.001; *: p<0.05

^a^Adjusted for year of survey, types of residence, and ecological zone.

^b^Adjusted for model 1; and wealth index, religion, ethnicity, mother’s education, mother’s literacy level, father’s education, mother’s occupation, mother’s age, mother’s desire for pregnancy, birth rank and birth interval, previous death of a child, and child sex.

^c^Adjusted for model 2; and types of drinking water source, types of sanitation facilities, and types of cooking fuel.
