# Supplementary material for: Under-Five Mortality and Associated Factors: Evidence from the Nepal Demographic and Health Survey (2001–2016)
Source: Int J Environ Res Public Health. 2019 Apr 8;16(7):1241. doi: 10.3390/ijerph16071241 (PMC6479835; doi:10.3390/ijerph16071241)
Supplement: Supplementary file 1 [file ijerph-16-01241-s001.zip › supplementary table 2.docx]

Supplementary table 2: aHR and 95% Confidence Interval (CI) for factors associated with child, and under-5 mortality in Nepal, 2001-2016 (15,750)

|  | Child mortality | | | Under-5 mortality | | |
| --- | --- | --- | --- | --- | --- | --- |
| Study Variable | Model 1^a^ | Model 2^b^ | Model 3^c^ | Model 1^a^ | Model 2^b^ | Model 3^c^ |
| Year of survey |  |  |  |  |  |  |
| 2001 | 1.00 | 1.00 |  | 1.00 | 1.00 | 1.00 |
| 2006 | 0.63(0.30, 1.31) | 0.64(0.31, 1.34) |  | 0.78(0.59, 1.03) | 0.86(0.66, 1.11) | 0.86(0.66, 1.11) |
| 2011 | 0.30(0.11, 0.86)* | 0.34(0.11, 1.02) |  | 0.65(0.48, 0.87)* | 0.82(0.61, 1.09) | 0.82(0.61, 1.09) |
| 2016 | 0.15(0.05, 0.45)* | 0.18(0.06, 0.59)* |  | 0.45(0.33, 0.63)** | 0.60(0.44, 0.83)* | 0.60(0.44, 0.83)* |
| Ecological zone |  |  |  |  |  |  |
| Terai |  |  |  | 1.00 | 1.00 | 1.00 |
| Hill |  |  |  | 0.91(0.72, 1.14) | 1.11(0.87,1.42) | 1.11(0.87,1.42) |
| Mountain |  |  |  | 1.48(1.11, 1.96)* | 1.45(1.08, 1.94)* | 1.45(1.08, 1.94)* |
| Ethnicity |  |  |  |  |  |  |
| Brahmin/chettri |  |  |  |  | 1.00 | 1.00 |
| Dalit |  |  |  |  | 1.25(0.91, 1.71) | 1.25(0.91, 1.71) |
| Janajati |  |  |  |  | 0.96(0.74, 1.24) | 0.96(0.74, 1.24) |
| Madhesi |  |  |  |  | 1.76(1.24, 2.49)* | 1.76(1.24, 2.49)* |
| Mother's literacy level |  |  |  |  |  |  |
| Can read |  |  |  |  | 1.00 | 1.00 |
| Cannot read |  |  |  |  | 1.53(1.18, 1.98)* | 1.53(1.18, 1.98)* |
| Mother occupation |  |  |  |  |  |  |
| Not working |  |  |  |  | 1.00 | 1.00 |
| Agriculture |  |  |  |  | 1.48(1.09, 2.00) | 1.48(1.09, 2.00) |
| Skilled/professional |  |  |  |  | 1.87(1.23, 2.84)* | 1.87(1.23, 2.84)* |
| Mother's age |  |  |  |  |  |  |
| 40-49 |  |  |  |  | 1.00 | 1.00 |
| 30-39 |  |  |  |  | 1.30(0.87, 1.92) | 1.30(0.87, 1.92) |
| 20-29 |  |  |  |  | 1.70(1.12, 2.57)* | 1.70(1.12, 2.57)* |
| <20 |  |  |  |  | 2.37(1.36, 4.16)* | 2.37(1.36, 4.16)* |
| Birth rank and birth interval |  |  |  |  |  |  |
| 2nd/3rd birth rank, >2 years |  | 1.00 | 1.00 |  | 1.00 | 1.00 |
| 1st child |  | 2.10(0.58, 7.61) | 2.11(0.62, 7.25) |  | 2.78(1.90, 4.07)** | 2.78(1.90, 4.07)** |
| 2nd/3rd child, interval ≤2 years |  | 1.35(0.41, 4.42) | 1.43(0.44, 4.68) |  | 1.19(0.80, 1.76) | 1.19(0.80, 1.76) |
| 4th/higher child, interval >2 years |  | 0.36(0.16, 0.83)* | 0.37(0.17, 0.84)* |  | 0.36(0.24, 0.53)** | 0.36(0.24, 0.53)** |
| 4th/higher child, interval ≤ 2 years |  | 0.87(0.40, 1.90) | 0.91(0.45, 1.87) |  | 0.62(0.43, 0.91)* | 0.62(0.43, 0.91)* |
| Previous Death of a child |  |  |  |  |  |  |
| No |  | 1.00 | 1.00 |  | 1.00 | 1.00 |
| Yes |  | 19.63(7.00, 55.08)** | 18.22(6.78, 48.95)** |  | 17.00(12.42, 23.28)** | 17.00(12.42, 23.28)** |
| Types of drinking water source |  |  |  |  |  |  |
| Improved |  |  | 1.00 |  |  |  |
| Unimproved |  |  | 3.94(1.29, 9.20)* |  |  |  |

aHR: adjusted Hazard Ratio; **: p<0.001; *: p<0.05

^a^Adjusted for year of survey, types of residence, and ecological zone.

^b^Adjusted for model 1; and wealth index, religion, ethnicity, mother’s education, mother’s literacy level, father’s education, mother’s occupation, mother’s age,

mother’s desire for pregnancy, birth rank and birth interval, previous death of a child, and child sex.

^c^Adjusted for model 2; and types of drinking water source, types of sanitation facilities, and types of cooking fuel.
